# Topics of Public Interest

**Published:** 1917-04

**Authors:** 


### TOPICS OF PUBLIC INTEREST

**Army Medical Corps.** Carl Scheffel of Brookline, Mass., Med. World, Mch., analyses the corps with regard to college of graduation. 83 colleges are represented, 35 extinct, the rest Class A except the National University, Class C, represented by a single captain. According to the 1916 American Medical Directory, the corps contains beside the surgeon-general, ranking as brigadier, 14 colonels, 24 lt. colonels, 104 majors, 228 captains, 49 1st lts. and 89 from the medical reserve corps on active duty: total 508. The routine promotion at the end of 4 years accounts for the preponderance of captains, along with the fact that only 11 candidates were commissioned in 1913, 15 in 1914 and 12 in 1915.

The statement that the medical reserve corps is to go out of existence this year, is literally correct but misleading. Officers of this corps will, if they desire, be transferred to the more general Officers' Reserve Corps up to June 3, 1917, when the former goes out of existence, and further appointments of medical reserve officers will be made. Really, instead of doing way with medical reserve officers, provision will be made for a still more efficient reserve. Commissions will be issued for 5 years, subject to re-examination and recommission. Attendance at camps of instruction for 15 days a year will be compulsory upon reserve officers, the government paying transportation and subsistence. They will also be compelled to take a correspondence course of instruction. Medical reserve officers above the grade of 1st lt. will not be liable to active duty in time of peace but 1st lts. will be. In war, any reserve officer may be ordered to active duty. A reserve officer cannot hold a commission in the National Guard.

The Surgeon General, U. S. A., announces that examinations for commission as first lt. in the active medical corps will be held on the first Monday of each month. There are at present 230 vacancies and after July 1, there will be 222 additional vacancies. Appointees made before July 1 will be ordered to the Army Medical School for a special session beginning July 9. The regular session begins Oct. 1. Applicants must be between 22 and 32, and a year's experience as interne is required but graduate physicians serving an interneship and who pass the examination, will be allowed to complete their interneship before going to the Army Medical School.

---

**National Wealth.** Estimated total 1916, 230 billion dollars, about $2,255 per capita, or $9,000 for each adult male. Of this, a little over 1% is in gold, minted or ready for coinage —$27 per capita, 2,741,000,000 in all. The total per capita money of all kinds is stated to be $43. (probably implying that paper money is counted only as actually backed by gold and silver deposits).

---

**New Hospital for Batavia.** The Sisters of Mercy of Buffalo have accepted the double house at 16 Bank St., bequethed by Miss Rose A. Jerome. It will be remodeled into a 50-bed hospital to be known as St. Jerome's.

---

**Keeping Intact the Practices of Volunteer Medical Officers in Case of War.** The suggestion is made by the Surgeon-General's Office that city, county and state medical societies formulate some plan by which, if the reserve medical corps is called into active service, the practices of the members may be kept intact and handed over to them after the expiration of their service. This is a good suggestion. Something after this plan was carried out during the Spanish-American War. It may be said, however, that there are certain practical obstacles to be considered. Such definite professional clienteles as formerly existed in this country and are, at least talked and written about, in England, have almost ceased to exist in many parts of this country. They certainly do not exist for surgeons and other specialists. The most dependable practices exist in small villages and isolated parts of some cities and are based on lack of competition rather than personal affiliation. In such instances, a newcomer entering the field would probably hesitate to do so merely as a locum tenens. In many instances, too, it might reasonably be expected that the physician for whom a practice was held, would never come back to it—not necessarily implying his death but his permanent attachment to the army, as happened in many cases in the Spanish-American War or his selection of surgery or choice of a different field of private practice. However, in spite of the impracticability of carrying out the suggestion in the literal sense in many cases, the spirit of the suggestion, that a man should not suffer unduly from competition arising on account of his patriotism, certainly should be observed. Possibly the government itself can co-operate, by providing that a medical officer, retained for a longer period than that of a fairly long vacation which would not affect his private practice seriously, shall not be discharged without other cause than cessation of hostilities, except after a rather long notice, or with the option of transfer to the regular service, or he might be allowed to compromise his period of notice by accepting part pay in advance, with immediate leave.

---

**To Complete Letchworth Village.** Joseph H. Choate, President of the State Charities Aid Assn. has asked the legislature for an appropriation of a million to complete the work at Thiells, Rockland Co. It is estimated that there are 33,000 feeble minded persons in N. Y. State outside of the city, of whom only 5,399 are in institutions intended for them while 4,500 are in jails, prisons, poor houses, etc. Of the 23,000 uncontroled, 6,600 are women of child-bearing age. Letchworth Village, as contemplated in 1907, was to care for 3,000 persons. At present, only 340 are provided for. Meantime, N. Y. City has increased its accommodations at Randall's Island from 778 to 2,021.

---

**The National Board of Medical** Examiners will hold its next examination in Washington during the week of June 13. Col., Del., Ia., Ky., Md., N. C., N. H., N. D., Pa. already recognize its certificate and favorable legislation is pending in 12 other states. It admits to the Army, Navy and the Graduate School of the University of Minn., including the Mayo Foundation.

---

**Requirements of Collegiate Work for Medical Licensure.** 1 year already operative: Conn., Kas., Utah, Vt. 2 years already operative: Col., Ind., Ia., Minn., N. D., S. D. 1 year, applying to graduates of 1918-21; no legislation as to 2 year requirements: Ark., Calif., Ill., Ky., Mich., Miss., N. C., Pa., Tenn., Tex., W. Va. 1 year applying to graduates of 1922, no legislation as to 2 year requirement: N. Y. 1 year applying to graduates of 1918-22, with requirement of 2 years by 1922: Ariz., Ia., Md., N. H., N. J., R. I., Va., Wash.

---

**Fly Catching.** Bull. 108 of the U. S. P. H. Service, details experiments by Earle B. Phelps and Albert F. Stevenson. Of sticky preparations, those containing castor oil are far superior to those containing other oils, white rosin being used in all cases in addition. 6-inch squares caught 35, 38 and 61 flies as compared with 0-8 on other spreads. Three commercial papers, not analysed, caught 25, 32 and 55 flies. Solutions of muscicides are considered somewhat superior and the following is advised as practically non-toxic to children and pets: 1% sodium salicylate; place in a tumbler, cover with a projecting sheet of white blotting paper, place a saucer or small plate on top and invert; a match inserted under the edge of the tumbler favors the feeding of the blotting paper with solution.

**Military Raw Material.** The N. Y. Committee on National Defense gives the following statistics: 30 million males in gainful occupations (the number of gentlemen of leisure in this country being negligible), of whom 21 million are between 18 and 45; 9 million unmarried; 10½ million physically fit for service. Note: Approximately, any country has 50% of its population male and 50% adult, about 20% being adult males of military age within wide limits. However, physical incapacity and the demands of industries that must be maintained even for war purposes, reduce the available militia to about 10%. In the Revolution and the Civil War, about 11% of the total population was enlisted, but not at any one time, and in the former the actual military service of the great majority was quite brief. For the four years of the Civil War, the total military deaths amounted to very nearly 10% of the forces or 1% of the population.

**The American Red Cross** reports a greater degree of efficiency than for any similar organization at any previous occasion, being able to care for an army of a million on a few days' notice. The organization consists of: 26 completely equipped army and navy base hospital units, with a total of 1250 nurses and 599 nurses' aids; a base reserve of 415 nurses and 525 nurses' aids; 31 partially complete navy detachments of 20 nurses each; 115 emergency detachments; 120 expert instructors in surgical dressings.

**County Tuberculosis Hospitals.** 33 of the 57 counties of the state outside of the city, have hospitals in operation or provided for. These contain 81.3% of the up-state population so that the urgency of the matter in the remaining 27 counties is obviously not so great. 27 hospitals are in use and 12 more authorized; 1799 beds available and 1,279 more authorized. When the state campaign began in 1907, there were 2 hospitals with 154 beds.

**The Tres Palacios Club.** This is being organized by Dr. Wm. F. Waugh of Palacios, Texas. It will occupy a square mile, and have 100 members, each owning 5 acres, the remainder being used for a club house, golf links, etc. The entire membership fee is $1000, payable in small installments. An artesian well supplies water, fruit trees of various kinds are to be planted and the 5 acre tracts should yield a considerable revenue. Various community privileges will be arranged as desired. The club will thus be available not only for vacations but for permanent residence and, solely from the economic standpoint should appeal especially to physicians wishing to retire.

---

**The Wicks-Grant Bill** introduced into the N. Y. legislature Feb. 15 is intended to carry out the joint report to the Governor, on Foods and Markets. It consolidates the present Depts. of Agriculture, Foods and Markets and Weights and Measures and transfers the functions of the Health Dept. relating to misbranding and adulteration of food, cold storage, etc. It gives added power to control food supplies, storage and distribution, to control competition and speculative prices, etc., and one of the functions of the new Dept. will be to publish information as to original, wholesale and retail prices. Ample power will be given to deal with such problems as have recently arisen in N. Y. City (and which, we may add, exist elsewhere, except that rioting has not resulted).

---

**Narcotic Convictions.** Edward Pring of Buffalo, has been convicted by a federal court of selling drugs and of employing agents for that purpose. He was sentenced to a "year and a day" in the Federal Prison at Atlanta.

---

**Advertising Physician Accused of Fraud by Labor Dept.** Dr. A. W. Bender who succeeded to Dr. Henry J. Shireson, now a fugitive from justice, in the conduct of a specialist's office at Main and Exchange Sts., Buffalo, was arrested Mch. 13. He is said to have worked particularly among ignorant foreigners, collecting advance fees for cures declared to be impossible without operation.

---

**A Bill to Repeal the Charter of the Rockefeller Foundation** has been introduced by Sen. John J. Boylan and Assemblyman Joseph M. Callahan.

---

**A Theft of Morphine Tablets,** presumably by an addict, was reported Mch. 13 by Dr. C. H. W. Auel of Buffalo.

---

**Fatal Ophiophobia.** Last May, a little girl threw a dead garter snake at Maggie Paonessa of Niagara Falls. She became violently hysterical and never fully recovered her health, dying Mch. 13. This is one of the most widely spread phobias, if not the normal state of man and is even shared by the lower animals to some degree. Serious nervous phenomena often develop from it and it should be respected by those who do not have it. Geo. H. Pepper of the Museum of the American Indian of New York City, in a recent lecture before the Buffalo Society of Natural Sciences, described the snake dances of the Hopis and stated that the Navajos attending the dances, took every occasion to spit on the snakes and the dancers as a token of their disgust though, in general, one tribe respects the religious rites of another. He also told of a white man, staying at a Navajo camp who handled a snake and afterward washed himself, using a piece of soap. An Indian woman picked up the soap on a stick and scraped off the surface before putting it back into its place. Most savages are relatively free from ophiophobia, at least in the literal aesthetic sense in which it is observed among more civilized peoples, though most tribes have myths and customs which plainly indicate the universal human tendency toward ophiophobia. It plays a part in most religions, even the Judaic and Christian. It may be remarked that a true -phobia is a horror or dread, not warranted by analysis. Thus photophobia is not included in this category of neuroses. Phthisiophobia though not necessarily warranted, is a fear of genuine danger and hence not a true neurotic -phobia. All true neurotic phobias are, however, based on genuine danger in the ultimate analysis. Whether they depend on hereditary memory or not is another question. Acrophobia, brontophobia, phobia, thalassophobia, arachnephobia, claustrophobia (the correct word not being in use) obviously are based on dangers which might be very real if it were not for circumstances. Ophiophobia is world-wide, according to evolutionists because, in the primitive life of man, the snake was the most insidious and therefore the most dangerous enemy, even creeping into arboreal retreats. While ophiophobia is perhaps even greater with regard to harmless snakes than toward larger, less sinuous poisonous snakes and constrictors, for which the realization of actual physical danger overrides the pure neurotic horror, it is interesting to note that all snakes are poisonous so far as their serum is concerned, even if lacking special glands for secreting the poison and apparatus for injecting it.

---

**Estimate of Total Militia of N. Y.** The total population is stated by the Health Dep't at 10,490,680, of whom 2,480,000 are males between 18 and 45. On the basis of rejections for the U. S. Army, the available militia is estimated at 570,000. (It should be remembered, however, that the requirements for the regular army in peace are rather higher than would be practical for war, and the class of applicants probably includes a disproportionate number of men undesirable for various reasons. We are inclined to believe that at least 2 million men could be raised in N. Y. State if necessary, and 20 million in the whole country.)

---

**Tuberculosis Clinics for Buffalo.** Patients will be examined and supplied with sputum cups, etc., as follows:

Health Center No. 1—Located at 1067 Grant street—District bounded by waterfront and Virginia, Niagara, Georgia, Chippewa, Main, Virginia, Delaware, Colvin, Kenmore, O'Neil to waterfront.

Health Center No. 2—591 William Street, corner Stanton—Bounded by William, Fillmore, Clinton, Jefferson, Swan, Pine, Broadway, Fillmore, Walden and city line.

Health Center No. 3—770 East Ferry Street—Bounded by city line and Walden, Fillmore, Broadway, Pine, Genesee, Chippewa, Main, Virginia, Delaware, Colvin, Kenmore and city line.

Health Center No. 4—404 Seneca Street (Welcome Hall)—Bounded by waterfront and Virginia, Niagara, Georgia, Chippewa, Genesee, Pine, Swan, Jefferson, Clinton, Fillmore, William, city line and waterfront.

Clinics will be held every Monday evening 7-8 at all centers, and at No. 1 Wed. 3.30-4.30; No. 2, Tues.; No. 3, Thurs. No. 4, Fri. at same hours. Emergent cases will be cared for on notice to the Div. of Tuberculosis, Dept. of Health. Mon. at 2 candidates for Perrysburg will be examined at 175 E. Swan street, after having applied for admission to this Division.

---

**Campaign Against Unclean Dairy Utensils.** The Dept. of Agriculture has plans for a home made sterilizer to be used with a kerosene stove, at a total cost of $15. This will be demonstrated for 2 weeks on application of local health and dairy officials. A comparative experiment showed that 5 gal. of fresh milk, using utensils washed in the ordinary way, yielded 1,880,000 bacteria per c.c., while the use of the home made sterilizer reduced the number to 24,000.

---

**Niagara Co. Tuberculosis Hospital.** Plans for a hospital on the pavilion plan have been submitted for approval.

---

**Legal Definition of Nurse.** The Mills bill would prohibit any but a training school graduate, registered by the Board of Regents, to use this designation. A representative of Catholic Hospitals has characterized this as an opportunity to trained nurses to form a trust.

**Compulsory Health Insurance.** At a recent hearing before the Senate Judiciary Committee, the Mills Bill was commended by some and opposed by others. For the bill were representatives of the Am. Assn. for Labor Legislation, State Charities Aid Assn., N. Y. Child Welfare League, International Ladies Garment Worker's Union, Columbia University and the N. Y. City Health Commissioner. Against were Dr. John D. Bonnar of the Med. Soc. of the Co. of Erie, and representatives of the N. Y. Chamber of Commerce, National Association of Manufacturers, State Federation of Labor and State Medical Society.

**Hair Snipper.** A man claimed to represent this type of sexual degeneracy has been arrested in Buffalo, after having snipped hair from several little girls. It should be borne in mind, however, that human hair has a commercial value and that this type of fetichism has been so much exploited in literature of a more or less romantic nature, that it often occurs without indicating actual sexual degeneracy. Somewhere, some years ago, we came across a young man who had a collection of pubic hair tied with ribbons. We do not remember who he was but have an impression that he was by no means a degenerate in the crude sense, though obviously not a model.

**Centenarians.** Justus Allen Record of Towanda, father of Dr. H. A. Record of Forestville, died Mch. 12. He was born Dec. 25, 1815. Mrs. Elizabeth Shively of Niagara Falls celebrated her 101st birthday Feb. 27 in the General Hospital, where she has been confined for 3 years on account of fractured hip. Mrs. Hessian Hollin of Richmond, Va., died recently in her 111th year, being considered the oldest woman in the state. Mrs. Alice Bennett of Flat Bush, N. Y., who was 105 years, old, Dec. 24, 1916, died Jan. 21.

**Animal Experimentation.** A bill has been introduced in the California Legislature providing that unclaimed cats and dogs in city pounds may be sold to universities and medical schools at 50 cents and one dollar, respectively, the animals to be kept in a sanitary manner and not to be subjected to surgical operation without full anaesthesia. This is a sensible, economic law, endorsed by G. H. Whipple Director of the Hopper Foundation for Medical Research, in the Mch. issue of the Calif. State Jour.

**Business Efficiency (?).** We have recently had shown us a bill to a semi-philanthropic institution, endorsed by six separate rubber stamps for approval and payment, these stamps covering all available space on both sides of the bill.

---

**Red Cross.** On Mch. 9, a smoker was given by Mr. Frank McGraw, Chairman of the Local Chapter, to the physicians of Buffalo, with a view to securing their co-operation.

